# Use of Single‐Cell Data and scPagwas Analysis to Identify T Cell Subsets and Construct a Prognostic Model for Clear Cell Renal Cell Carcinoma

**DOI:** 10.1155/humu/1916444

**Published:** 2026-04-08

**Authors:** Xincheng Yi, Zongming Jia, Jixiang Wu, Siyu Wang, Yiqi Yu, Ying Kong, Xuefeng He, Yuhua Huang

**Affiliations:** ^1^ Department of Urology, The First Affiliated Hospital of Soochow University, Suzhou, China, sdfyy.cn; ^2^ Zhongshan School of Medicine, Sun Yat-sen University, Guangzhou, China, sysu.edu.cn

**Keywords:** clear cell renal cell carcinoma, DOCK8, scPagwas, single-cell data analysis, T cell

## Abstract

**Background:**

Clear cell renal cell carcinoma (KIRC), the most prevalent pathological renal cell carcinoma (RCC) subtype, makes up approximately 75%–84% of total cases. KIRC is characterized by high heterogeneity, high metastasis rates, and a poor prognosis. Its incidence rate has continued to rise in recent years. We sought to construct new prognostic models to optimize treatment decisions, improve clinical benefits, and explore potential therapeutic targets.

**Methods:**

This study integrated various omics data including single‐cell RNA seq (GSE171306), TCGA‐KIRC, GWAS, and validation datasets (GSE29609 and E‐MTAB‐1980). The scPagwas algorithm combines GWAS with scRNA‐seq to identify immune subgroups with high feature correlation. Key genes are identified through the combination of weighted correlation network analysis (WGCNA) with differentially expressed genes (DEGs). We built a clinical prognostic model by using machine learning algorithms and validated it through survival rate and receiver operating characteristic (ROC) analysis. We used cancer drug sensitivity genomics data to analyze drug sensitivity and performed molecular docking to identify potential therapeutic drugs.

**Results:**

Using single‐cell RNA seq data, we identified T cell subsets as characteristic cell subsets in KIRC through scPagwas analysis. In single‐cell analysis, key genes in T cell subsets and genes with PCC values > 0.05 were combined with the core genes in DEGs and WGCNA modules, thus yielding 86 intersecting genes. These genes were significantly enriched in immune‐related pathways. We established a clinical prognostic model containing seven risk genes. Low‐risk patients exhibited substantial survival advantages. Time‐dependent ROC analysis indicated the prognostic model′s excellent clinical predictive value. Functional enrichment, immune infiltration, and somatic mutation analyses highlighted different biological mechanisms among risk populations. The SHAP values of the XGBoost and LightGBM machine learning algorithms indicated DOCK8 as a potential biomarker. Drug prediction and molecular docking predicted five potential drugs targeting DOCK8 (finasteride, nocodazole, palonosetron, pifithrin alpha, and topiramate).

**Conclusion:**

Our systematic analysis of the immune microenvironment, key genes, and prognosis of KIRC highlighted the critical roles of T cell subsets. We additionally established an effective clinical prognostic model. Our findings provide new insights and potential targets for the precise diagnosis and targeted KIRC therapy.

## 1. Introduction

Renal cancer is the 10th‐most prevalent mortality cause among men and the 11th‐most prevalent mortality cause among women. Renal cell carcinoma (RCC) is highly invasive and associated with elevated mortality, and is a common reproductive and urinary tumor type [1, 2]. Clear cell renal cell carcinoma (KIRC), the most common RCC pathological subtype, accounts for approximately 75%–84% of all cases [3]. KIRC exhibits high heterogeneity, high metastasis rates, and poor prognosis [4]. In recent years, the incidence rate of KIRC has continued to rise. Although early or localized cases can achieve favorable prognosis through surgical resection, approximately 25% of localized cases show recurrence after surgery, thus highlighting the major challenges in clinical management [5, 6]. Despite growing treatment options for KIRC, given its complex biological characteristics, patients still face key issues such as drug resistance, inadequate heterogeneity management, and low long‐term survival rates. Immunotherapy with immune checkpoint inhibitors (ICIs) has substantially greater efficacy than typical cytokine‐based therapy. Nevertheless, prolonged use can lead to drug resistance and consequently affect treatment effectiveness [7]. Therefore, constructing new prognostic models may aid in the selection of better treatment options according to patient conditions, maximization of clinical benefits, and support for target mining, thereby advancing precision medicine.

The tumor microenvironment (TME) has essential roles in tumor presence, metastasis, and progression. Its main components include stromal cells, tumor cells, immune cells, extracellular matrix, and vascular endothelial cells. These components, through complex interactions, collectively form a dynamic microenvironment that substantially influences tumor development [8]. KIRC′s interaction with the TME is central to tumor evolution, invasion, treatment response, and metastasis [9, 10]. Therefore, KIRC progression is associated with an abnormal TME, and TME changes accelerate RCC occurrence and progression by promoting immune escape [11]. In the TME, CD8+ T cells, CD4+ T cells, and NK cells have antitumor effects by clearing tumor cells [12]. However, tumor cells may suppress immune cell activity by secreting inhibitory cytokines and decreasing or altering antigen expression on the tumor surface, thereby decreasing the likelihood of recognition as foreign by the immune system. In addition, tumor cells induce immune tolerance by recruiting immunosuppressive cells, which evade immune surveillance and clearance mechanisms [13–17]. The extracellular matrix provides important structural support for tumor cells by promoting cell adhesion and infiltration. Moreover, tumor cells reshape the extracellular matrix by secreting proteolytic enzymes, thereby enhancing their invasion and metastasis capabilities [18]. Although the TME is essential in tumor development, its multidimensional heterogeneity remains to be fully analyzed. Determining how to efficiently integrate multiple omics data to comprehensively reveal the complex relationship between TME and KIRC, and further explore precise therapeutic targets and develop innovative therapies, remains an important current research direction. Future research combining cutting‐edge techniques such as single‐cell sequencing and machine learning is urgently needed to systematically elucidate the potential biological functions and clinical significance of TME in KIRC, thus providing a theoretical basis for guiding the development of precision treatment strategies.

The scPagwas method, a pathway‐based multigene regression framework, projects trait‐related genetic variations into cellular pathways and generates trait‐related scores (TRSs) quantifying the association between the gene expression profiles of various cell populations and unsupervised deep imaging phenotype‐related genetic effects, thereby further bridging the gap between genome‐wide association study (GWAS) outcomes and single‐cell data [19]. We used the scPagwas method to project KIRC GWAS information onto single‐cell data for evaluating TRSs of specific cell subpopulations. We also explored the functional mechanisms of these cell subpopulations in KIRC, aiming to identify potential novel biomarkers.

## 2. Materials and Methods

### 2.1. Data Gathering and Processing

TCGA‐KIRC data obtained from the TCGA database contained mRNA expression and related clinical data from 72 normal samples and 542 KIRC samples. We downloaded the single‐cell dataset GSE171306 and the GEO dataset GSE29609 from the GEO database (https://www.ncbi.nlm.nih.gov/geo/). The E‐MTAB‐1980 dataset was downloaded from the ArrayExpress database. The GSE171306 dataset contains sequencing information for two KIRC tissues. GSE29609 and E‐MTAB‐1980 served as the validation set. Furthermore, summary information (ukb‐b‐1316) of a KIRC GWAS came from the comprehensive epidemiological unit OpenGWAS (https://gwas.mrcieu.ac.uk/) and included 1114 cases, 461,896 controls, and 9,851,867 single nucleotide polymorphisms.

### 2.2. Analysis of Single‐Cell KIRC Data

We first analyzed the single‐cell KIRC dataset GSE171306. We performed quality control by screening cells with < 200 expressed genes or > 5000 expressed genes, as well as mitochondrial gene content exceeding 10%. The “FindVariable Features” function and “vst” identified the top 2000 highly variable genes in cells, and outcomes are depicted in the form of a scatter plot. To aid in assessment and investigation of cell groups, we used the “JackStraw” and “JackStrawPlot” functions to perform linear dimensionality reduction according to each cell′s gene expression, then used the “FindNeighbors” and “FindClusters” functions to conduct cell clustering analysis according to a decreased cell expression matrix, thus clarifying the data structure. Finally, we annotated cells from various subgroups by using the “SingleR” software package [20].

### 2.3. ScPagwas Analysis of scRNA‐Seq and GWAS Data

To quantify the associations between certain genetic features and each cell or cell population, we used the scPagwas algorithm to merge single‐cell transcriptome data with GWAS findings. This complex computational tool [19] enables isolation of immune cell subpopulations that might contribute to heritable characteristics by computing a TRS. This technology helps accurately identify gene variants that influence the immunological response and other biological processes and represents a major step toward understanding disease causes and identifying treatments. The scPagwas software program was used to determine the TRS of every cell type. The immune cells with the highest TRSs were then selected for further examination.

### 2.4. Screening of Weighted Correlation Network Analysis (WGCNA) and Differentially Expressed Genes (DEGs)

In the TCGA‐KIRC queue, we analyzed differential expression between normal and KIRC samples to screen for DEGs. We used the “limma” package from R with logfc = 1 and a threshold of *p* < 0.05 after correction. We also used the “WGCNA” software package to fit and establish gene coexpression networks associated with immune cell subpopulations [21]. We selected the top 50% genes with the largest differences between samples as the input matrix for subsequent WGCNA. We calculated the Pearson correlation coefficient between genes and determined a soft threshold of 14 to ensure that the gene network conforms to a scale‐free topology. This selection is based on the evaluation of the fitting index (R^2^) and average connectivity of the scale‐free topology model. Subsequently, a hierarchical clustering tree diagram was generated, where different branches represent different gene modules. Genes with similar expression patterns are divided into modules, each module containing at least 30 genes. Subsequently, similar modules were combined at a cutting height threshold of 0.25. *p* < 0.05 indicates a significant correlation between this module and the status of immune cell subpopulations. Further analysis was conducted on the modules with the highest correlation coefficients and their core genes.

### 2.5. Screening of Prognosis‐Related Genes

We performed intersection analysis on the core genes of feature subgroups obtained from single‐cell analysis, DEGs, core genes in WGCNA related modules, and trait‐related genes ranked according to PCC to identify the intersecting genes. To further understand these intersecting genes′ possible roles, we conducted Gene Ontology (GO) analysis to investigate molecular functions, biological processes, and cellular components [22]. Gene set enrichment analysis and Kyoto Encyclopedia of Genes and Genomes (KEGG) analysis were used to identify pathways in which these genes might potentially be involved [23, 24]. Subsequently, on the basis of the intersecting genes, we used univariate Cox regression analysis to screen prognosis‐associated genes.

### 2.6. A Comprehensive Machine Learning Algorithm for Construction of a KIRC Clinical Prognostic Model

To develop a highly stable and accurate clinical prognostic model, we integrated 10 machine learning algorithms and 101 of their combinations. The algorithms were elastic network (Enet), CoxBoost, Least Absolute Shrinkage and Selection Operator (Lasso), generalized boosted regression modeling (GBM), Ridge, partial least squares regression for Cox (plsRcox), random survival forest, supervised principal components (SuperPC), survival support vector machine (survival‐SVM), and stepwise Cox. A leave‐one‐out‐cross‐validation (LOOCV) framework was applied to avoid overfitting and to produce an optimally stable clinical prognostic model [25]. Finally, the consistency index (c‐index) was applied to assess model performance and select the optimal clinical prognostic model.

### 2.7. Evaluation and Validation of Clinical Prognostic Models

In the TCGA‐KIRC queue as well as two validation sets (GSE29609 and E‐MTAB‐1980), patients were classified as either low risk or high risk according to their median risk scores. The “survminer” R package was used to establish survival curves corresponding to the OS. The time‐dependent ROC curve was used to assess model accuracy. Univariate and multivariate Cox analyses based on the clinical prognostic model′s clinical attributes and risk scores were conducted to determine the risk factors for clinical outcomes in patients with KIRC.

### 2.8. Nomogram Construction

To identify survival duration‐related risk scores and clinical factors, we applied univariate and multivariate Cox proportional hazards analyses. Subsequently, we constructed a nomogram integrating prognostic features and used it to predict 1‐, 3‐, and 5‐year OS in patients with KIRC.

### 2.9. Correlation Between the Clinical Prognostic Model Risk Score and Immune Cell and Somatic Cell Mutations

We used the CIBERSORT algorithm to determine tumor infiltrating immune cell abundance in all samples [26]. Subsequently, patients were classified as either low risk or high risk according to the median risk scores, and the group differences in tumor infiltrating immune cell populations were examined. The “maftools” software package was used to assess somatic mutation differences between groups.

### 2.10. Immunohistochemistry

Paraffin‐embedded specimens were sectioned into 4‐*μ*m–thick slices for immunohistochemical staining. First, sections were dewaxed in xylene and rehydrated via a graded ethanol series. To block endogenous peroxidase activity, we incubated sections with 3% H_2_O_2_ at room temperature for 15 min, then rinsed them with PBS. Nonspecific binding sites were then blocked with 5% BSA at 37°C for 1 h. After blocking, sections were incubated overnight at 4°C with primary antibodies (82370‐2‐RR, Proteintech). Immunohistochemical staining was subsequently performed with an SP Rabbit & Mouse HRP Kit (DAB) (CWBIO, China) according to the method of Jia et al. The method′s description partly reproduces their wording [27].

### 2.11. Ethical Approval

Between January 2024 and April 2025, we surgically obtained a series of six paired cancer and adjacent nontumor tissue samples from patients diagnosed with KIRC at The First Affiliated Hospital of Soochow University (Table S1). All specimens were histopathologically validated postsurgery, and no enrolled patients had received antitumor treatment before surgery. The research protocol was reviewed and approved by the Institutional Ethics Committee of The First Affiliated Hospital of Soochow University (Approval Number 2025‐732). Written informed consent was obtained from each participant before sample collection.

### 2.12. Drug Sensitivity Analysis

We downloaded relevant information from the cancer drug sensitivity genomics database (https://www.cancerrxgene.org/). The “oncoPredict” package from R was used to predict each tumor sample′s drug sensitivity. The Wilcoxon test was used to assess drug sensitivity differences between groups [28].

### 2.13. Determination of Important Features and Molecular Docking

We used the machine learning algorithms LightGBM and XGBoost to assess the feature importance of model genes to identify key genes contributing most in the model. To explain how each feature affected the model′s output, we determined SHAP values. To identify genes with major effects on the model′s overall output, we averaged each gene′s SHAP value across all samples. On the basis of the most strongly contributing genes, we identified DEGs between the low‐ and high‐expression groups. Subsequently, using the Connectivity Map database (cMAP, https://portals.broadinstitute.org/cmap/), we further investigated possible DEG‐based therapeutic drugs [29]. We used the PubChem database to obtain small molecule 3D structures in saf format. The AlphaFold Protein Structure Database was used to obtain 3D structures of protein structures and save them in pdb format. We used the online CB‐Dock2 database (http://clab.labshare.co.uk/cb-dock/php/index.php) for molecular docking alongside complete visualization analysis.

### 2.14. Statistical Analysis

R Version 4.3.3 was used for all statistical analysis and visualization. For comparisons between two groups of samples, we applied the Wilcoxon test, whereas for comparisons of multigroup differences, we used the Kruskal–Wallis test and one‐way analysis of variance. The Benjamini–Hochberg method was used to correct the *p* value. The threshold for statistical significance was *p* < 0.05.

## 3. Results

### 3.1. Identification of Immune Cell Subsets in KIRC

First, we conducted quality control analysis on the single‐cell KIRC dataset. Figure 1a shows the correlation between sequencing depth and gene expression, mitochondrial gene percentage, and ribosomal gene percentage. Figure 1b shows the gene expression distribution, sequencing depth, mitochondrial gene proportion, and ribosomal gene percentage. The UMAP algorithm served as a dimensionality reduction method to divide all cells into 23 cell clusters (Figure 1c). Finally, we annotated 23 cell clusters into seven cell subgroups by searching for relevant genes with the CellMarker database (Figure 1d). Figure 1e shows the proportion of cell subpopulations among samples.

Figure 1Recognition of immune cell subpopulations in KIRC. (a) Correlation between sequencing depth and gene expression level, including percentages of mitochondrial genes and ribosomal genes. (b) Distribution of gene expression levels, sequencing depth, and percentages of mitochondrial and ribosomal genes in the sample. (c) The UMAP algorithm displayed 23 cell subpopulations. (d) A total of 23 cell subpopulations were annotated as seven cell subpopulations. (e) Proportions of cell subpopulations in each sample.

**Figure:**
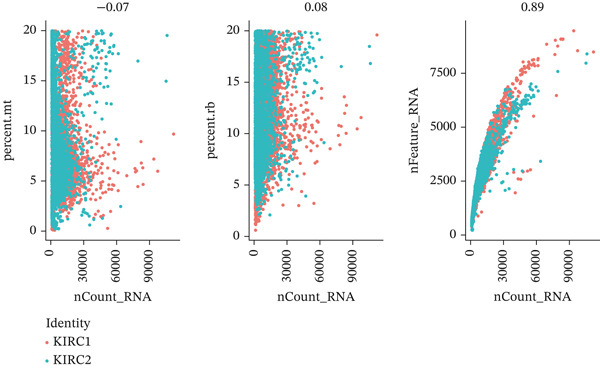
(a)

**Figure:**
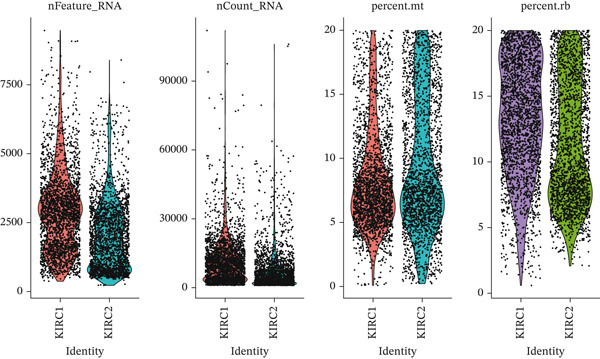
(b)

**Figure:**
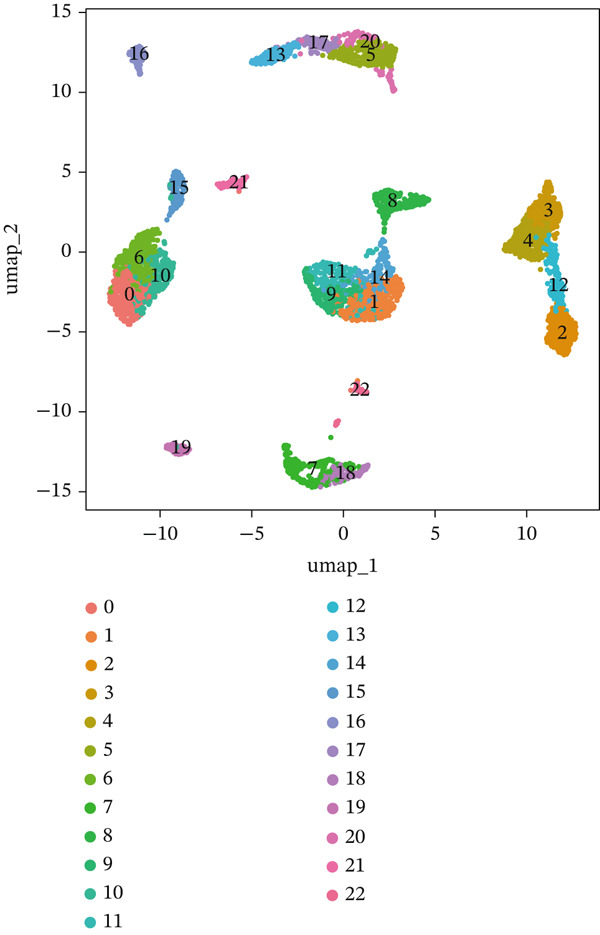
(c)

**Figure:**
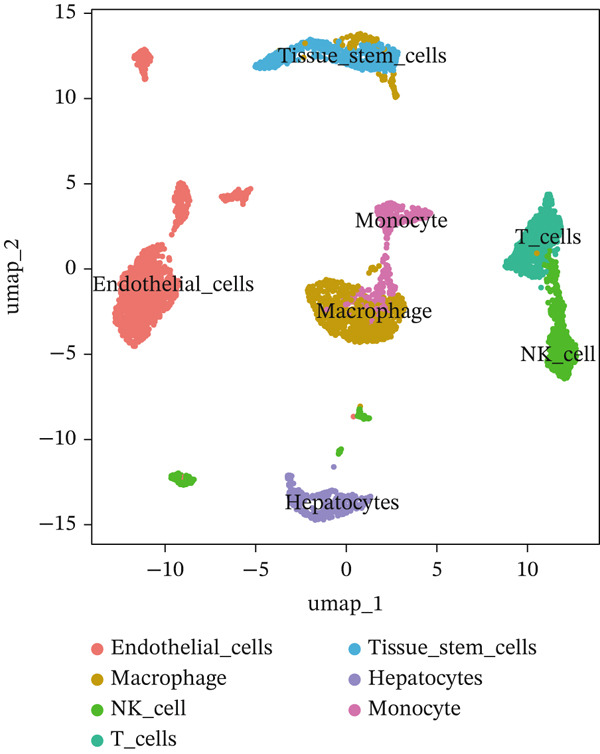
(d)

**Figure:**
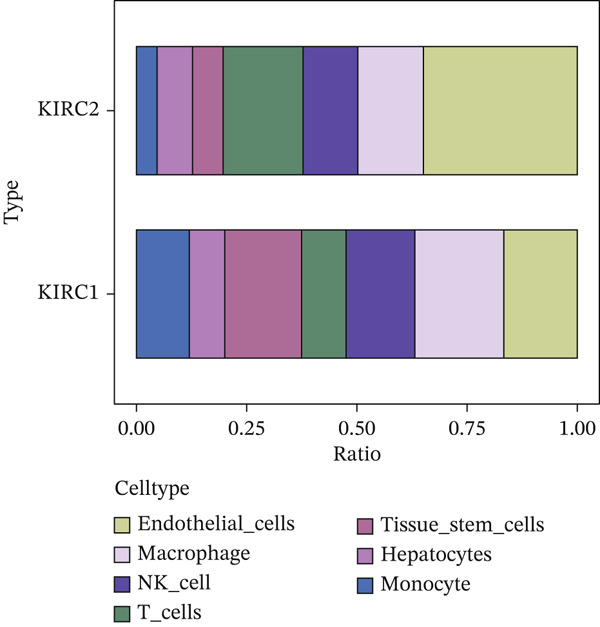
(e)

### 3.2. scPagwas Identifies Characteristic Cell Populations Associated With KIRC

We integrated single‐cell datasets containing KIRC and GWAS datasets, then applied scPagwas to identify immune cell subpopulations associated with KIRC. The T cell subset in KIRC had the highest TRS and showed significant differences alongside other cell subsets (Figure 2a). We further determined the top trait genes associated with KIRC by calculating the correlation between specific gene expression and each cell′s overall genetic pleiotropy association score (gPAS) (Figure 2b). Genes with PCC values > 0.1 were included in subsequent analysis.

Figure 2scPagwas identifies the cell subpopulations most relevant to KIRC. (a) Left: UMAP diagram showing the distribution of cell subpopulations. Middle: Distribution of cell subpopulations according to scPagwas. Right: Differences in TRS scores among cell subpopulations. (b) Heat map depicting Pearson correlation coefficients (PCCs) of gene expression profiles. Genes are sorted by hierarchical clustering, with color levels representing PCC values.

**Figure:**
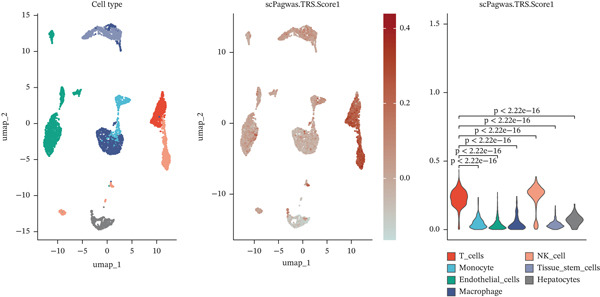
(a)

**Figure:**
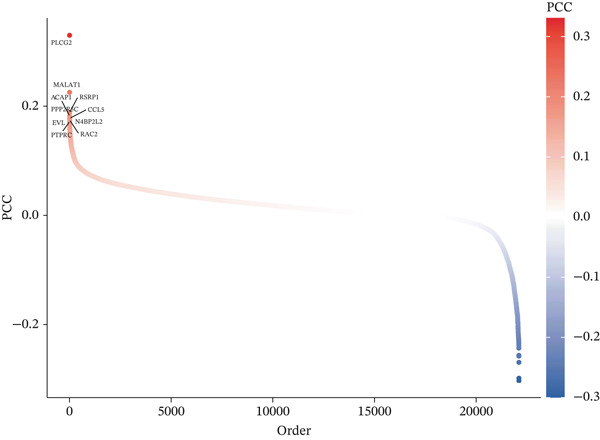
(b)

### 3.3. Differential Expression Analysis and WGCNA Analysis in TCGA‐KIRC

In the TCGA‐KIRC queue, the “limma” package was used to analyze differential expression between KIRC samples and normal samples, and 19,774 DEGs were obtained. Heat maps and volcano plots were used to visualize DEGs (Figure 3a,b). WGCNA analysis was performed to identify key gene modules associated with T cell subsets in KIRC. On the basis of average connectivity and scale independence, we set 14 as the soft threshold (Figure 3c). We obtained 13 modules, each with similar gene coexpression characteristics (Figure 3d). We evaluated the modules′ relationships with T cell subsets by determining correlations between ME values and T cell subsets. The magenta (*r* = 0.45, *p* = 2*e* − 31), blue (*r* = 0.44, *p* = 9*e* − 31), and cyan modules (*r* = 0.42, *p* = 3*e* − 28) significantly correlated with the T cell subsets (Figure 3e). Finally, 3628 core genes were included in subsequent analysis.

Figure 3Differential expression analysis and WGCNA analysis. (a) Heat map displaying DEGs between KIRC samples and normal samples. (b) Volcano plot displaying DEGs between KIRC samples and normal samples. (c) The soft threshold was set to 14. (d) Tree diagram of 13 modules marked with different colors. (e) Correlations of the magenta module, blue module, and cyan module with T cell subpopulations.

**Figure:**
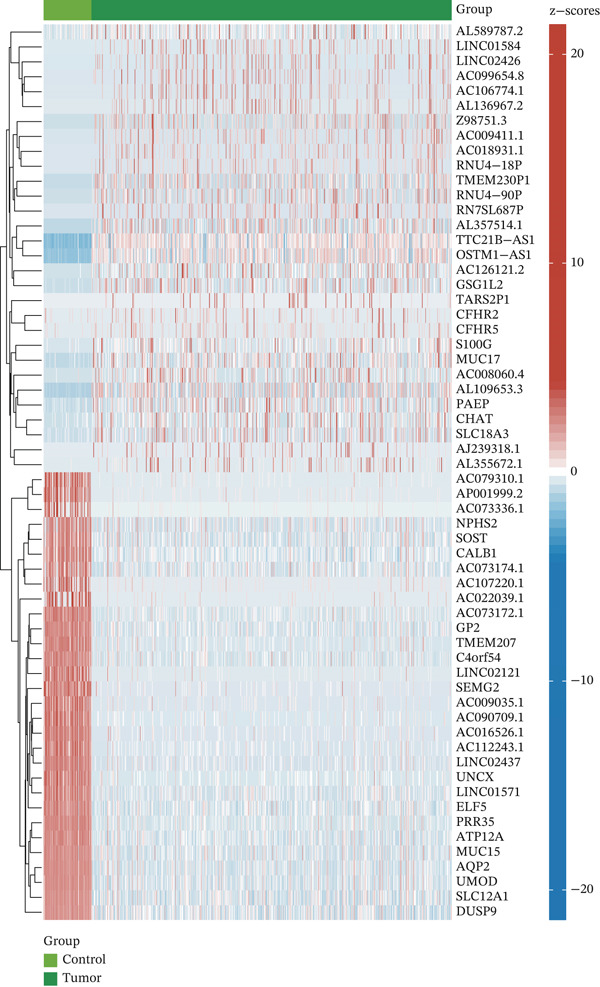
(a)

**Figure:**
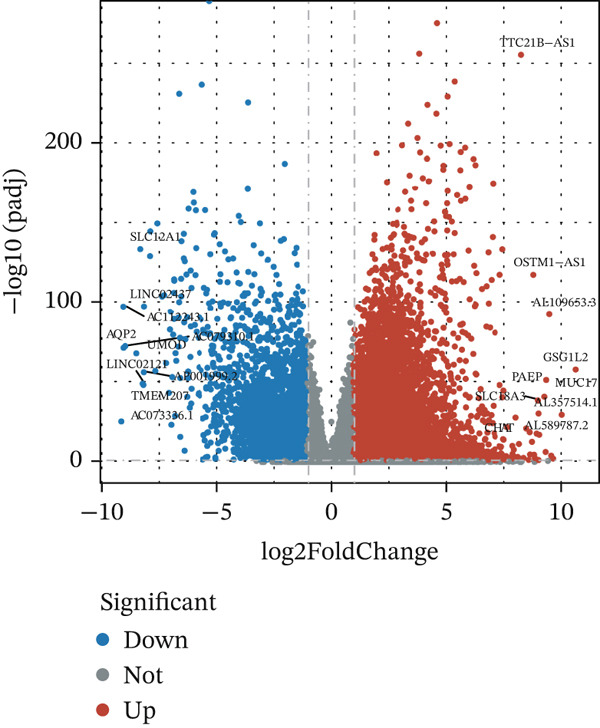
(b)

**Figure:**
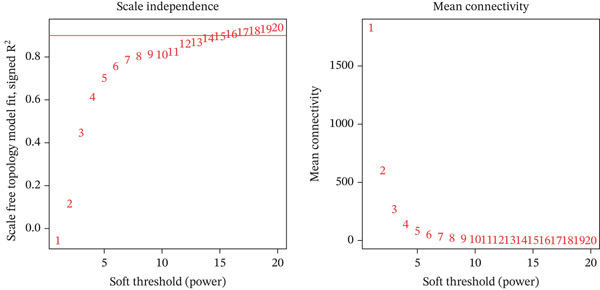
(c)

**Figure:**
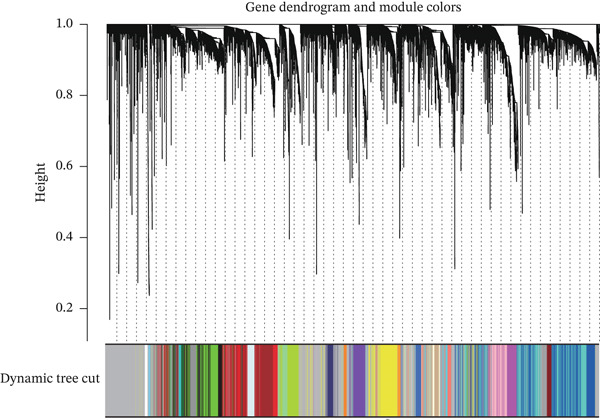
(d)

**Figure:**
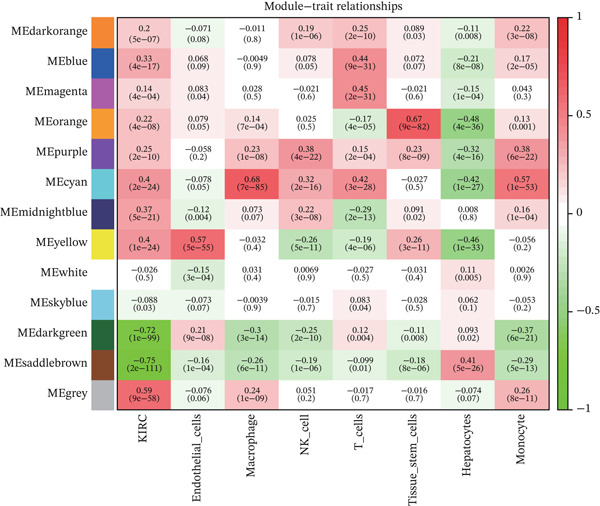
(e)

### 3.4. Identification of Prognosis‐Associated Genes

We conducted intersection analysis on key genes of T cell subsets, DEGs, core WGCNA module genes, and genes with PCC values > 0.05 in a single‐cell analysis. We obtained a total of 86 intersecting genes (Figure 4a). To identify possible mechanisms associated with these intersecting genes in KIRC, we performed KEGG and GO analyses. The GO analysis results indicated that these intersecting genes were closely associated with the occurrence and regulation of immune responses, such as the activation and regulation of lymphocytes and white blood cells, white blood cell adhesion, and differentiation of lymphocytes and monocytes (Figure 4b). KEGG analysis outcomes indicated that these genes were closely associated with adaptive and innate immunity, signal transduction, infection, transplant immunity, metabolic regulation, and immune escape, in pathways including transplant rejection, the NOD‐like receptor signaling pathway, the TNF signaling pathway, the NF‐*κ*B signaling pathway, and other biological pathways (Figure 4c). Finally, to identify prognostic genes, we conducted univariate Cox regression analysis, which identified 54 genes.

Figure 4Identification of prognostic genes. (a) Venn diagram displaying 86 intersecting genes. (b) GO analysis of intersecting genes. (c) KEGG analysis of intersecting genes.

**Figure:**
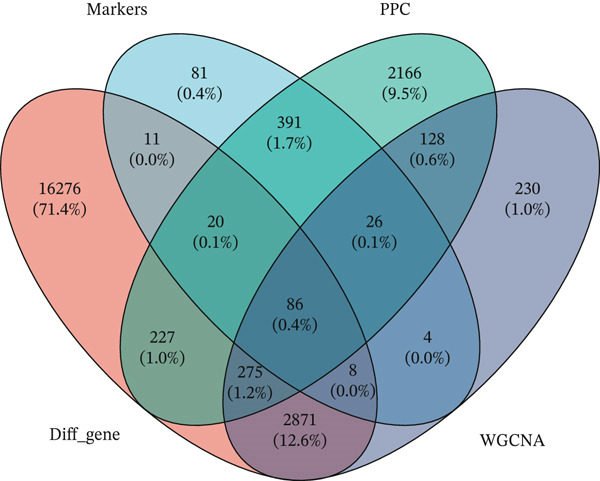
(a)

**Figure:**
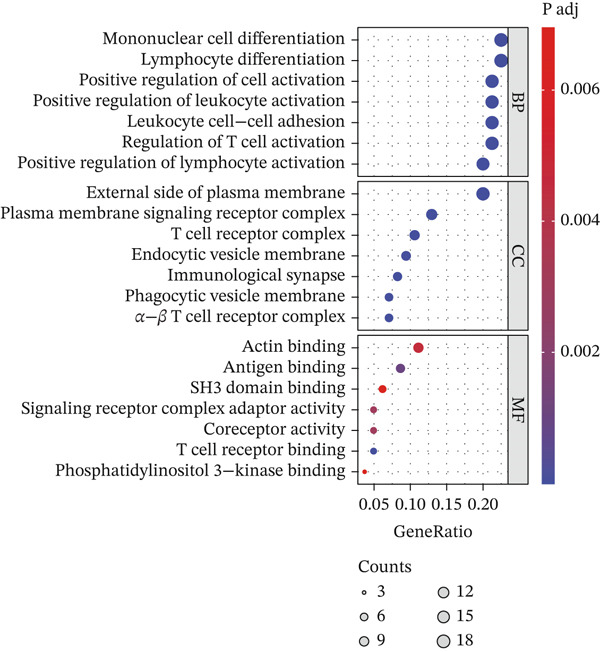
(b)

**Figure:**
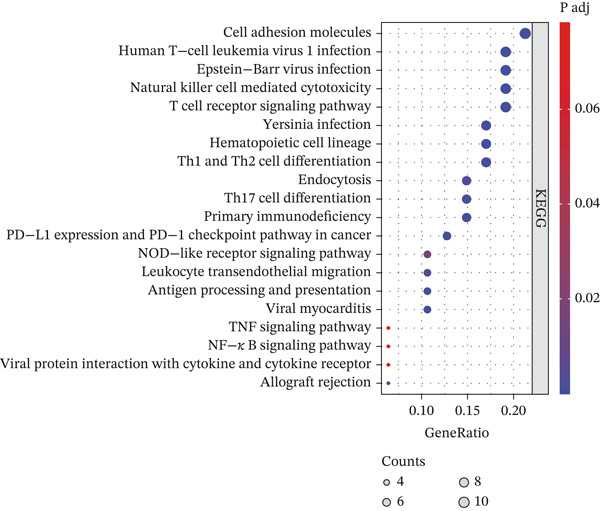
(c)

### 3.5. Construction of a Clinical Prognostic Model Associated With T Cells in KIRC

For the TCGA‐KIRC dataset, we fitted prediction models for 101 combinations with the LOOCV framework. Each model′s C‐index was determined. Notably, the combination with the greatest C‐index was random survival forest, and the C‐index was 0.766 (Figure 5a). Finally, we identified a clinical prognostic model with seven key genes (CASP4, CCM2, CCNL2, donor of cytokines 8 [DOCK8], LENG8, PABPN1, and TCIRG1). Subsequently, we divided the patients in the training (TCGA‐KIRC) and validation sets (GSE29609 and E‐MTAB‐1980) into high‐ or low‐risk groups according to the clinical prognostic model′s median risk value. Higher risk scores of clinical prognostic models were substantially associated with adverse clinical outcomes in patients with KIRC (Figures 5b, 5c, and 5d). Survival analysis outcomes indicated that the low‐risk group of patients with KIRC showed a significant survival advantage (Figures 5e, 5f, and 5g). ROC analysis was performed to evaluate the clinical prognostic model′s value. Evaluation of the area under the time‐dependent curve indicated that the clinical prognostic model exhibited high accuracy in predicting the clinical results of patients in all KIRC cohorts. In the TCGA‐KIRC queue, the AUCs for 1, 2, and 3 years were 0.965, 0.978, and 0.984, respectively. In the GSE29609 queue, the AUCs were 0.665, 0.622, and 0.688, respectively. In the E‐MTAB‐1980 queue, the AUCs were 0.682, 0.703, and 0.734, respectively (Figures 5h, 5i, and 5j). Furthermore, univariate and multivariate Cox regression analyses revealed that the clinical prognostic model′s risk score served as an independent risk factor for clinical outcomes in patients with KIRC (Figure 5k,l).

Figure 5Construction and validation of the clinical prognostic model. (a) Evaluation of the C‐index of prediction models generated through LOOCV in the TCGA‐KIRC, GSE29609, and E‐MTAB‐1980 queues. (b–d) Survival status and time distribution of risk scores for clinical prognostic models in the TCGA‐KIRC, GSE29609, and E‐MTAB‐1980 cohorts. (e–g) Survival analysis between high‐ and low‐risk groups in the TCGA‐KIRC, GSE29609, and E‐MTAB‐1980 cohorts. (h–j) ROC analysis of clinical prognostic models in the TCGA‐KIRC, GSE29609, and E‐MTAB‐1980 cohorts. (k–l) Univariate and multivariate Cox regression analysis of risk scores in the clinical prognostic model. (k) Univariate Cox regression analysis. (l) Multivariate Cox regression analysis.

**Figure:**
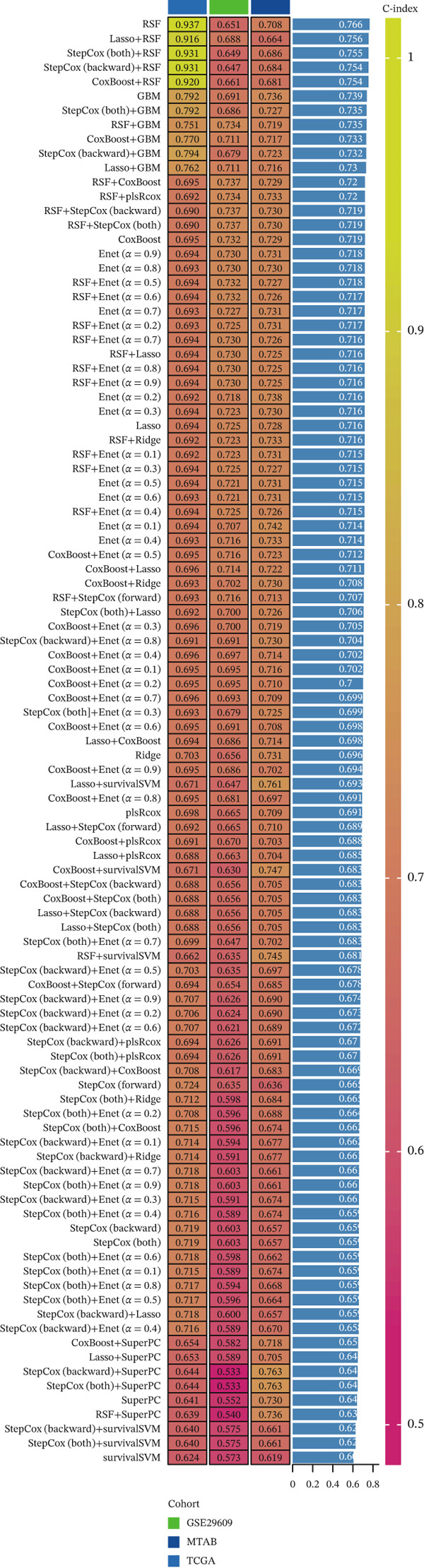
(a)

**Figure:**
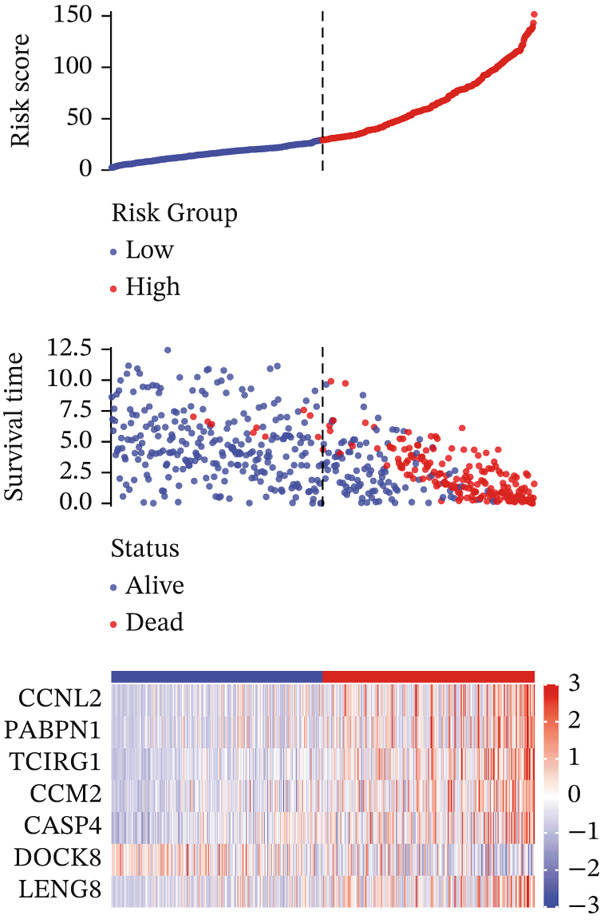
(b)

**Figure:**
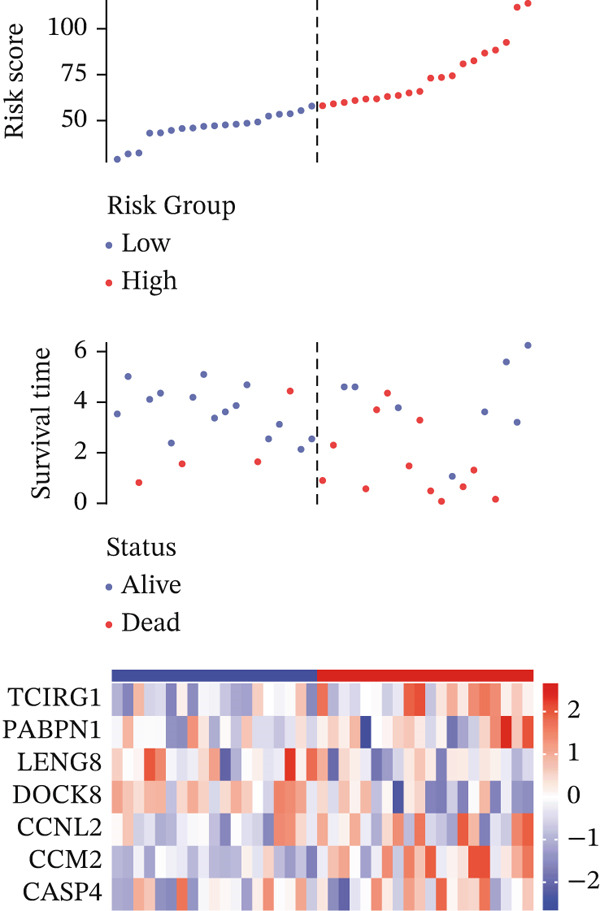
(c)

**Figure:**
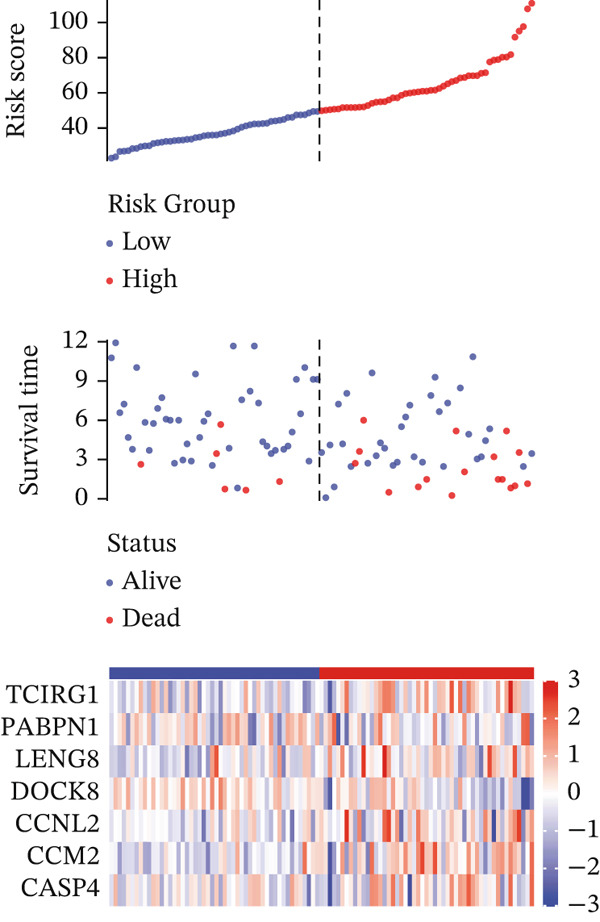
(d)

**Figure:**
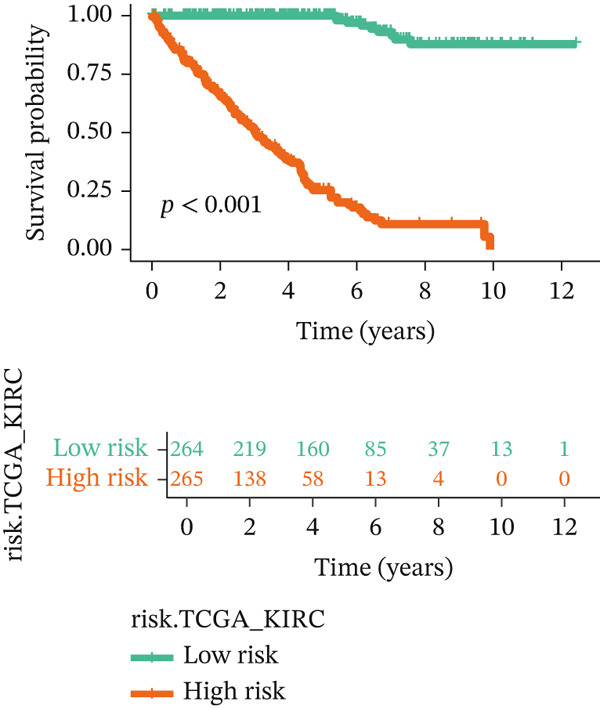
(e)

**Figure:**
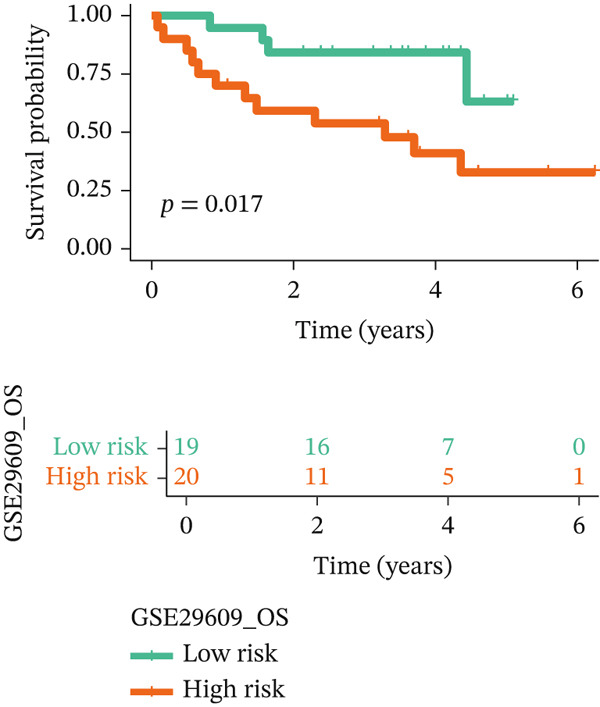
(f)

**Figure:**
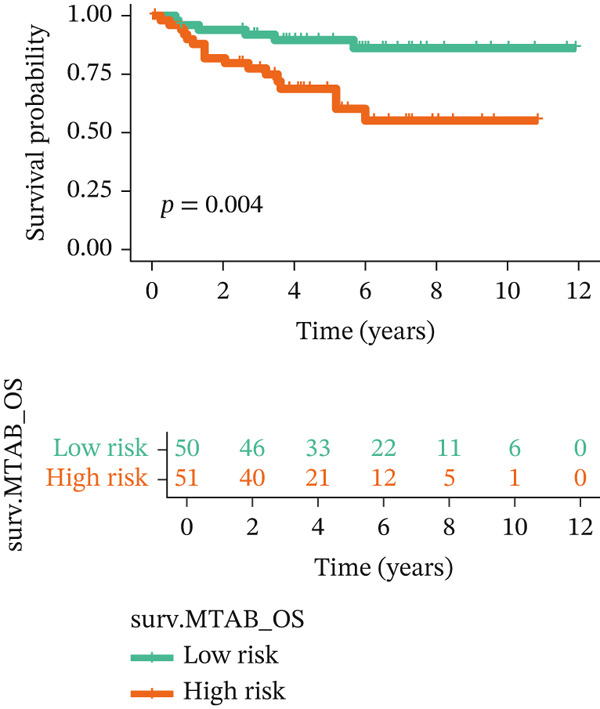
(g)

**Figure:**
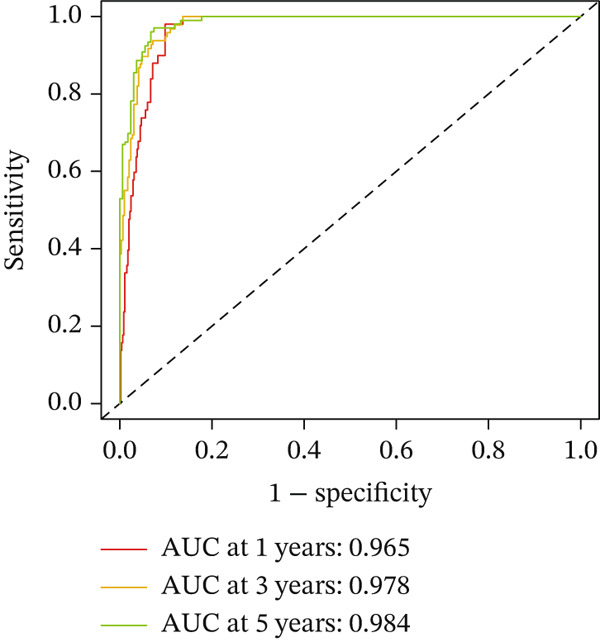
(h)

**Figure:**
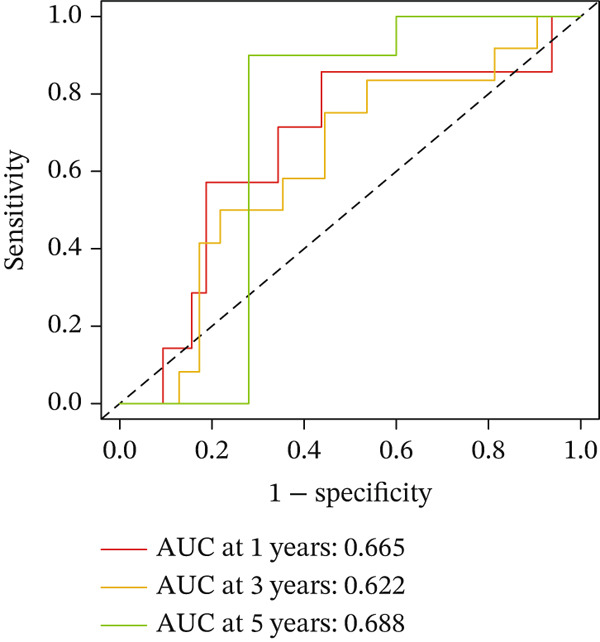
(i)

**Figure:**
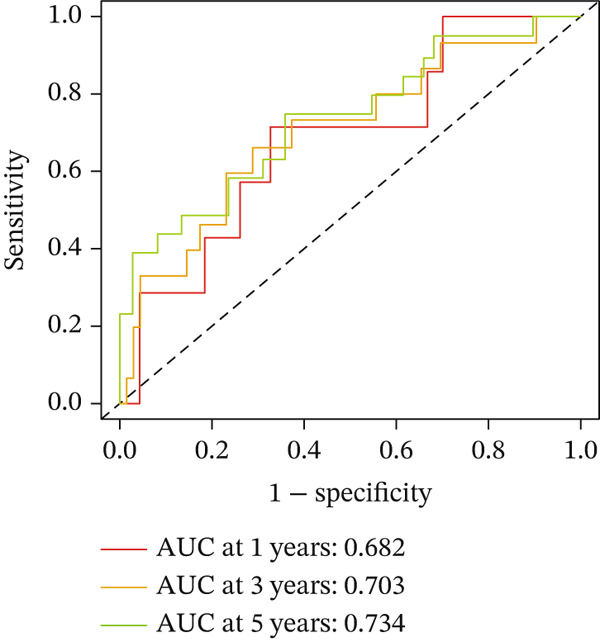
(j)

**Figure:**
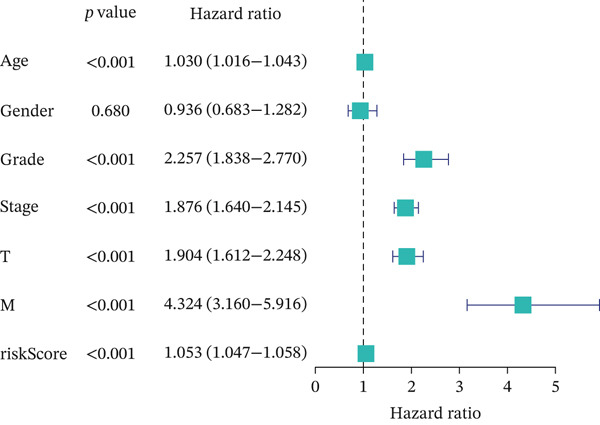
(k)

**Figure:**
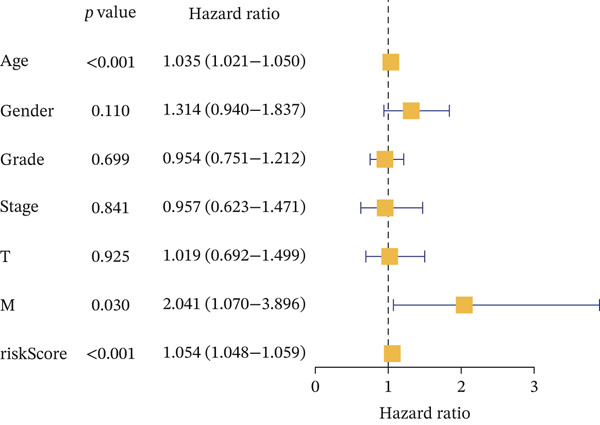
(l)

### 3.6. Nomogram Construction and Verification

To improve the model′s practicality in clinical decision‐making and enable a more comprehensive approach to predicting prognosis in patients with KIRC, we developed a nomogram combining risk scores alongside clinical features (sex, age, stage, grade, and TM staging). We rigorously validated the prediction accuracy of the nomogram with a calibration curve comparing the predicted survival probability with the observed results (Figure 6a). The high degree of consistency indicated that our nomogram accurately reflected patient survival rates (Figure 6b). Risk accumulation graph outcomes indicated that the cumulative risk significantly increased with time among high‐risk patients (Figure 6c).

Figure 6Development of a new nomogram associated with KIRC. (a) Development of a nomogram combining clinical features and risk scores (∗: *p* < 0.05; ∗∗: *p* < 0.01; and ∗∗∗: *p* < 0.001). (b) Nomogram calibration curves for 1, 2, and 3 years. (c) Risk accumulation chart, stratified by risk.

**Figure:**
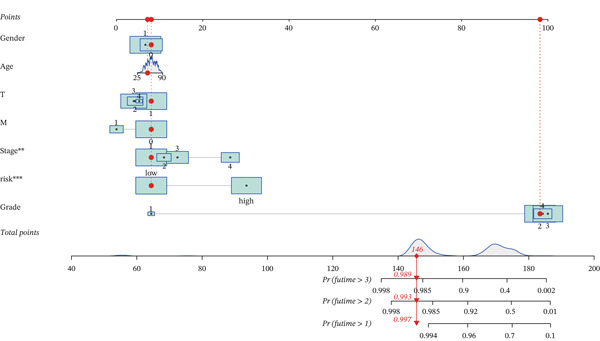
(a)

**Figure:**
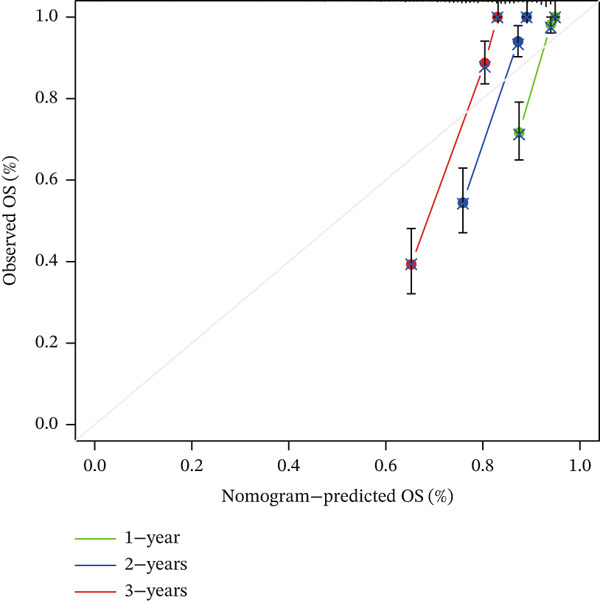
(b)

**Figure:**
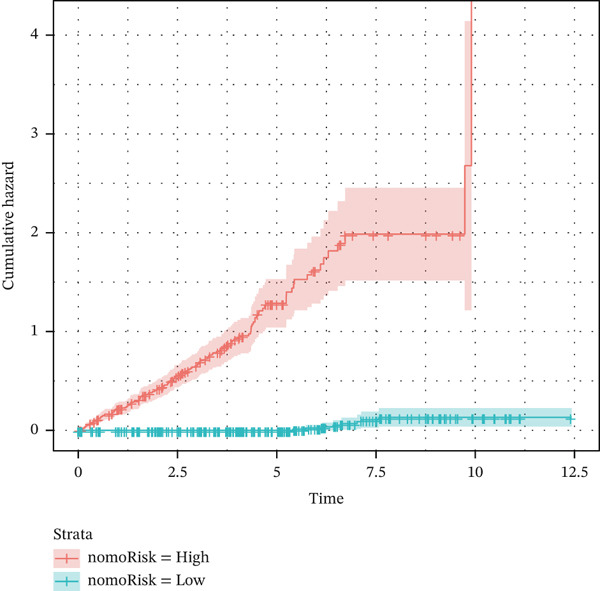
(c)

### 3.7. Functional Enrichment Analysis Between the Low‐ and High‐Risk Groups

To explore possible biological mechanisms underlying group differences, we conducted gene set enrichment analysis to identify significantly enriched KEGG pathways. Metabolic pathways such as those associated with fatty acid, propionic acid, and retinol were significantly enriched in the low‐risk group (Figure 7a). Cell adhesion molecules, dilated cardiomyopathy, ECM–receptor interactions, and other pathways were significantly enriched in the high‐risk population (Figure 7b).

Figure 7Functional enrichment analysis. (a) KEGG pathways enriched in the low‐risk population. (b) KEGG pathways enriched in the high‐risk population.

**Figure:**
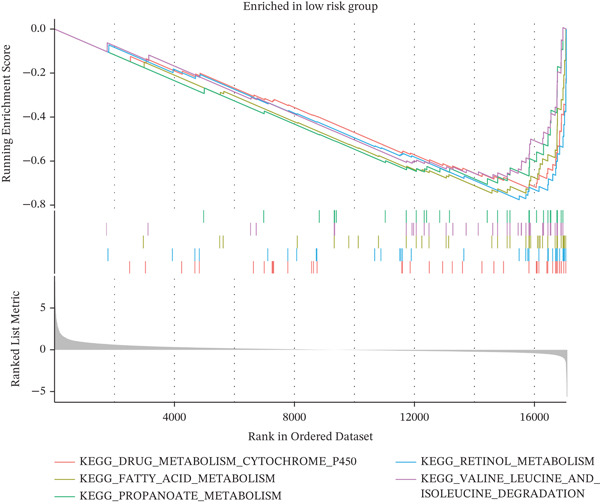
(a)

**Figure:**
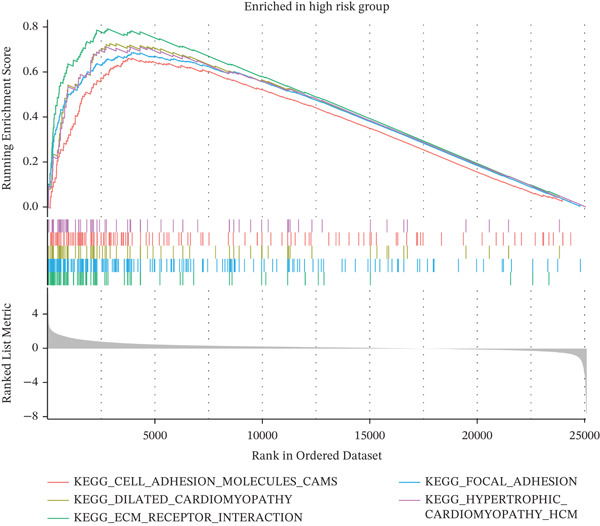
(b)

### 3.8. Relationship of Risk Score With Immune Cell Infiltration and Mutation Status

Substantial differences in immune infiltration were observed between the low‐ and high‐risk groups. CD4 memory activated T cells, plasma cells, M0 macrophages, regulatory T cells (Tregs), and follicular helper T cells were substantially upregulated in the high‐risk group. Significant restoration of CD4 memory T cells, dendritic cells, M1 macrophages, and mast cells was observed in the low‐risk group (Figure 8a). A correlation analysis between risk score and immune checkpoint proteins indicated that the risk score was positively associated with ATIC, CTLA4, and PDCD1, and negatively correlated with OLA1 and HAVCR2 (Figure 8b). We observed a substantial difference in tumor mutational burden (TMB) between the low‐ and high‐risk groups (Figure 8c). A waterfall plot depicted the mutations of the 20 most common genes in the two groups. In the low‐risk cohort, approximately 81.5% of the samples showed genetic mutations (Figure 8d), whereas in the high‐risk population, approximately 82.14% of the samples showed mutations (Figure 8e).

Figure 8Correlations of the risk score with immune cell infiltration and mutation status. (a) Differences in immune infiltration between samples from the low‐ and high‐risk groups. (b) Correlations between the risk score and immune checkpoint proteins. (c) Differences in TMB levels between the low‐ and high‐risk groups. (d) Waterfall diagram of genetic mutations in the low‐risk population. (e) Waterfall diagram of genetic mutations in the high‐risk population.

**Figure:**
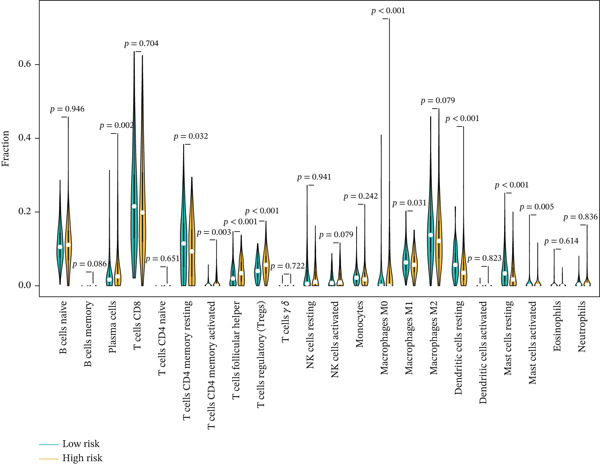
(a)

**Figure:**
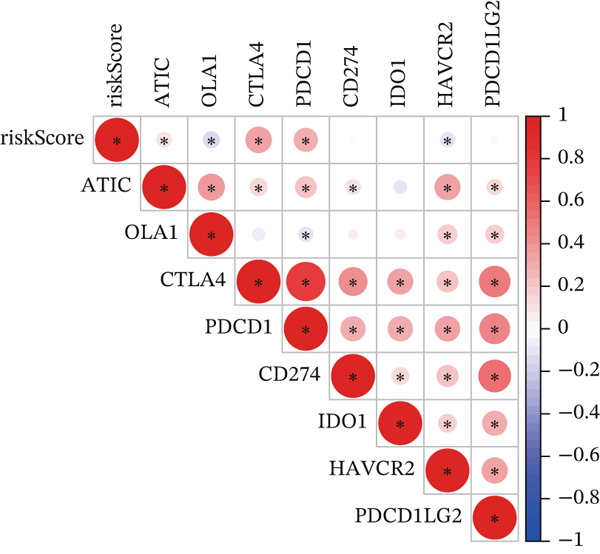
(b)

**Figure:**
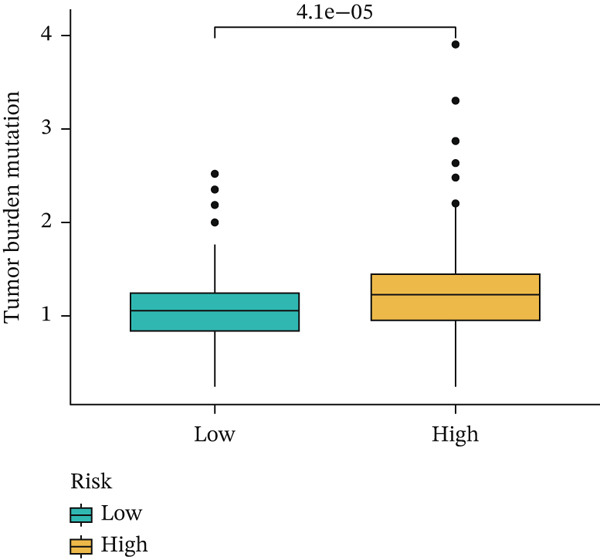
(c)

**Figure:**
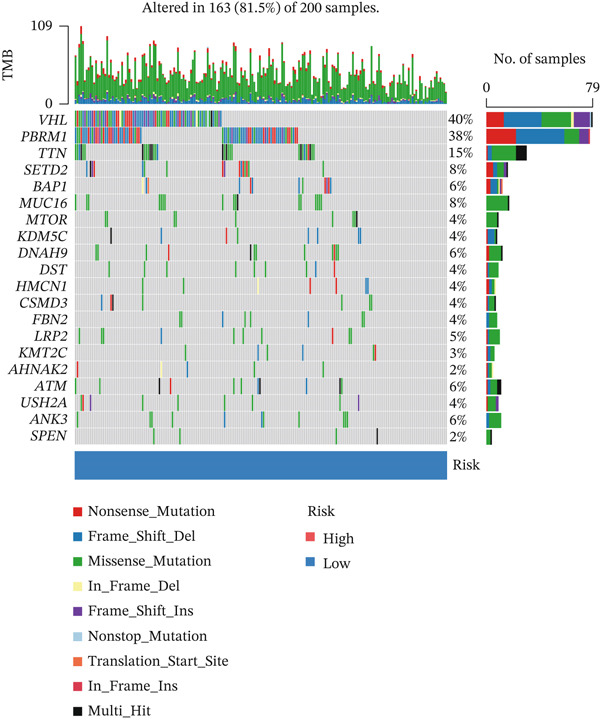
(d)

**Figure:**
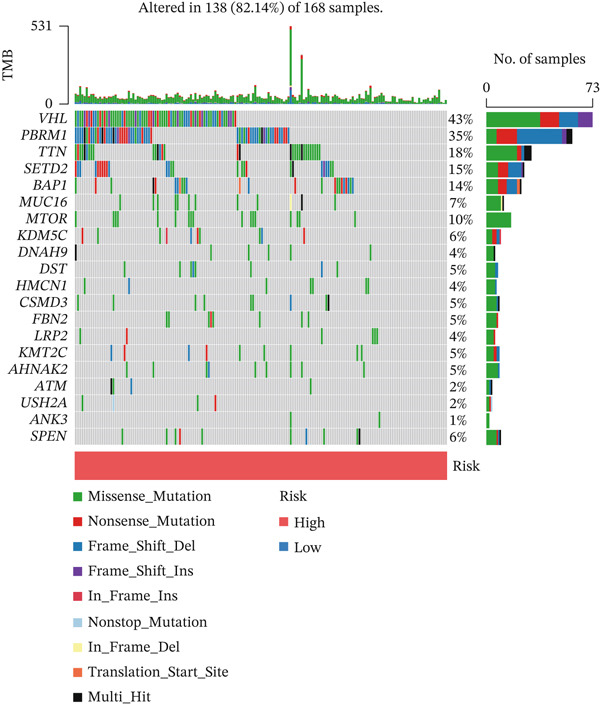
(e)

### 3.9. Drug Sensitivity

We next conducted drug sensitivity analysis to screen anti‐tumor drugs for various risk populations. Crizotinib, lapatinib, linsitinib, nilotinib, and vinblastine had higher IC50 values in the low‐risk group than in the high‐risk group (Figures 9a, 9b, 9c, 9d, and 9e). This indicates that high‐risk patients are aware of such drugs. In contrast, ibrutinib, osimertinib, and RO‐3306 exhibited lower IC50 values in the low‐risk group than in the high‐risk group (Figures 9f, 9g, and 9h), thus indicating the sensitivity of low‐risk patients to these drugs.

Figure 9Drug sensitivity analysis. (a) Crizotinib. (b) Lapatinib. (c) Linsitinib. (d) Nilotinib. (e) Vinblastine. (f) Ibrutinib. (g) Osimertinib. (h) RO‐3306.

**Figure:**
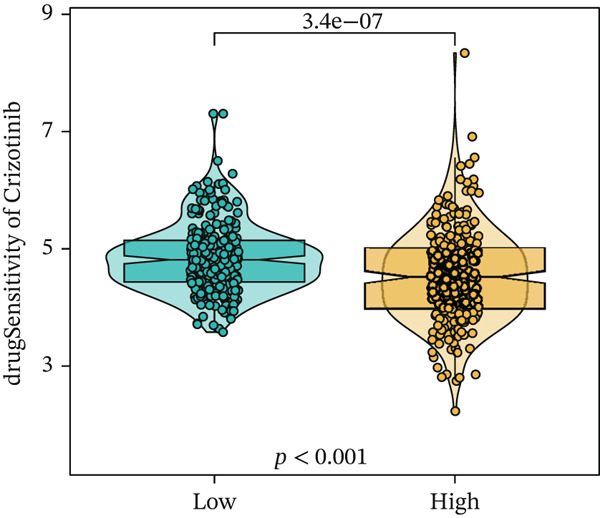
(a)

**Figure:**
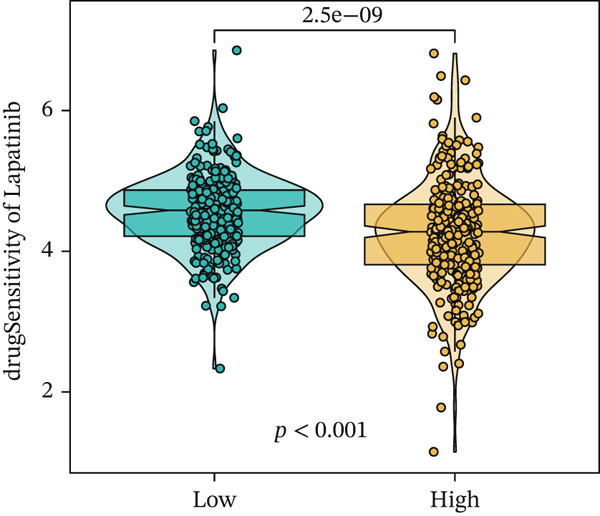
(b)

**Figure:**
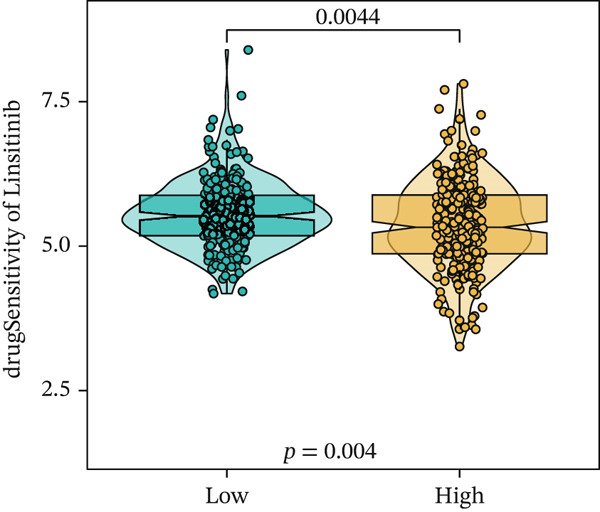
(c)

**Figure:**
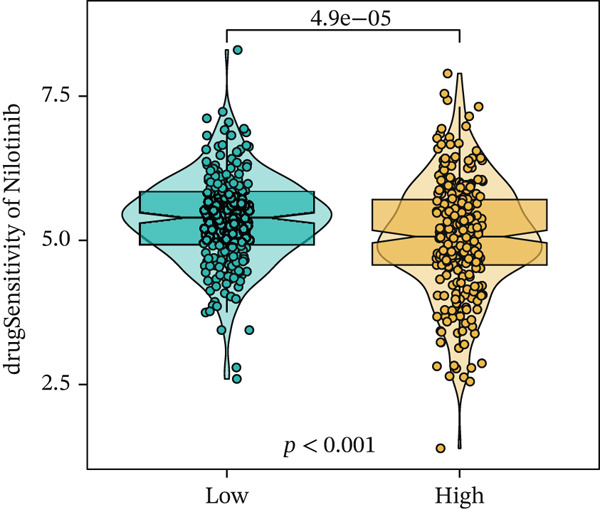
(d)

**Figure:**
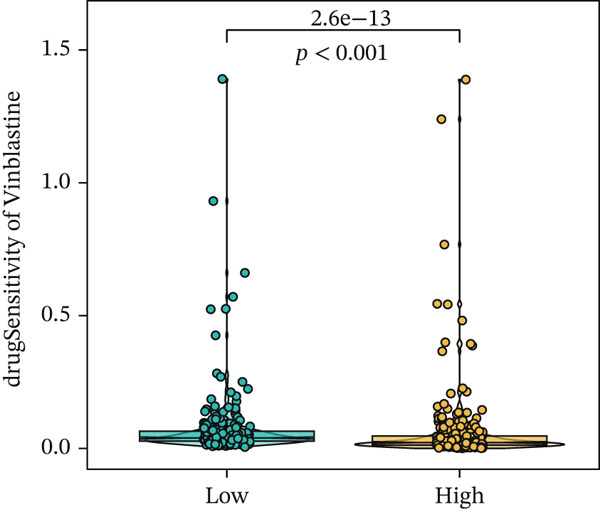
(e)

**Figure:**
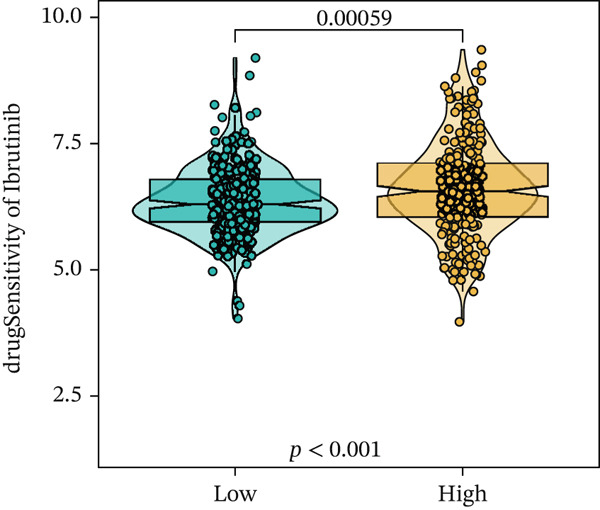
(f)

**Figure:**
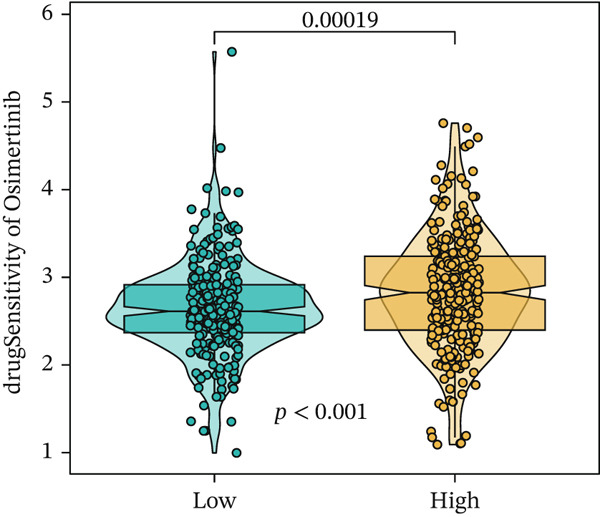
(g)

**Figure:**
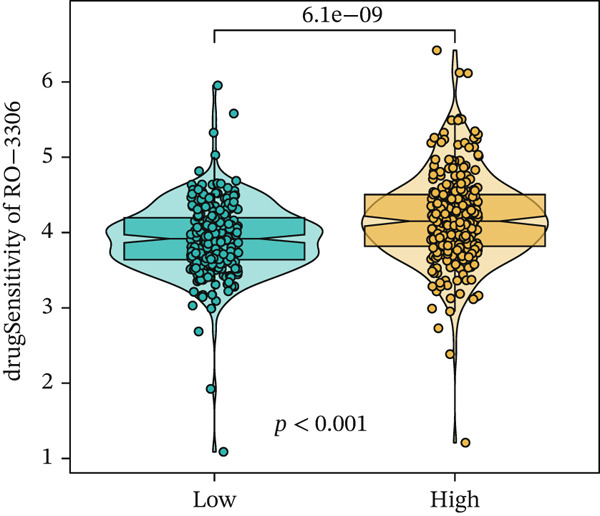
(h)

### 3.10. Identification of Important Risk Genes in Clinical Prognostic Models, Drug Prediction, and Molecular Docking

We analyzed the importance of seven risk genes (CASP4, CCM2, CCNL2, DOCK8, LENG8, PABPN1, and TCIRG1) in the model by using LightGBM and XGBoost, and sought to explain each gene′s involvement in the model through SHAP values (Figure 10a,b). The risk genes are listed in descending order of their effects on model output. DOCK8 ranked first in the two algorithms, thus indicating its stable and significant effects on the model. Therefore, we considered DOCK8 to be a key gene and subjected it to subsequent analysis. To examine the effect of DOCK8 on KIRC, we performed immunohistochemistry assays on KIRC tissues and adjacent normal renal tissues, and observed higher DOCK8 expression in tumor tissues (Figure 10c). To predict small molecule drugs targeting DOCK8, we divided the TCGA‐KIRC cohort into high or low expression groups, then conducted differential expression analysis. The upregulated DEGs can be downloaded from the cMap database. Analysis of connectivity scores identified five targeted drugs (finasteride, nocodazole, palonosetron, pifithrin alpha, and topiramate). To better analyze the most effective small molecule drugs targeting the DOCK8 protein, we conducted molecular docking analysis between the DOCK8 protein and the top five screened drugs. Tighter conformational binding was indicated by lower binding energies. Excellent binding was indicated by a binding energy below −5 kcal/mol, whereas high activity was indicated by a binding energy below −7 kcal/mol. All small molecule therapeutic candidates bound the target protein DOCK8 with energies below −6 kcal/mol. The binding energy was −8.0 kcal/mol for finasteride, −8.0 kcal/mol for nocodazole, −8.8 kcal/mol for palonosetron, −8.3 kcal/mol for pifithrin alpha, and −8.0 kcal/mol for topiramate (Figures 10d, 10e, 10f, 10g, and 10h).

Figure 10Identification of important risk genes in clinical prognostic models, drug prediction, and molecular docking. (a–b) Use of XGBoost (a) and LightGBM (b) machine learning algorithms to calculate the contribution value of each gene to the model, sorted from maximum to minimum. The SHAP value represents the absolute average effect of each gene on the model. (c) Immunohistochemical staining of DOCK8 in KIRC tissues and adjacent normal renal tissues. Quantitative analysis was performed in ImageJ software (∗: *p* < 0.05; ∗∗: *p* < 0.01; and ∗∗∗: *p* < 0.001). (d–h) Binding sites of ligands in proteins, demonstrating interactions between ligands and residues. The docking method was structure based blind docking. (d) Finasteride. (e) Nocodazole. (f) Palonosetron. (g) Pifithrin alpha. (h) Topiramate.

**Figure:**
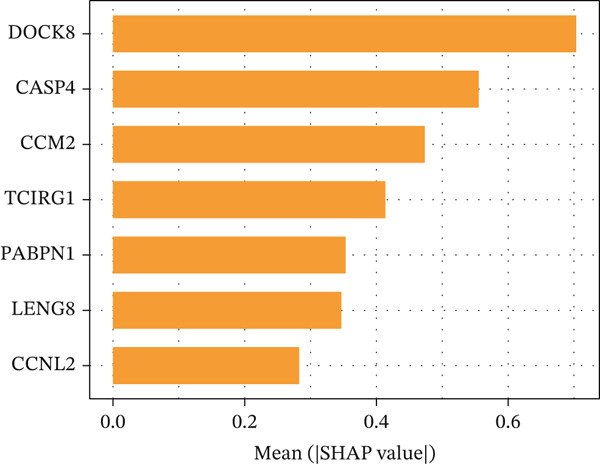
(a)

**Figure:**
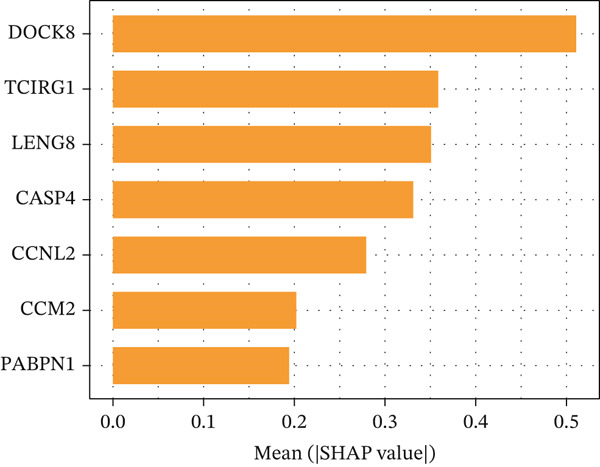
(b)

**Figure:**
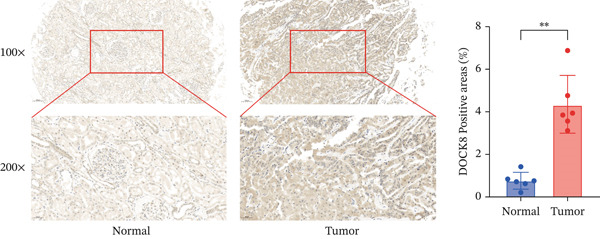
(c)

**Figure:**
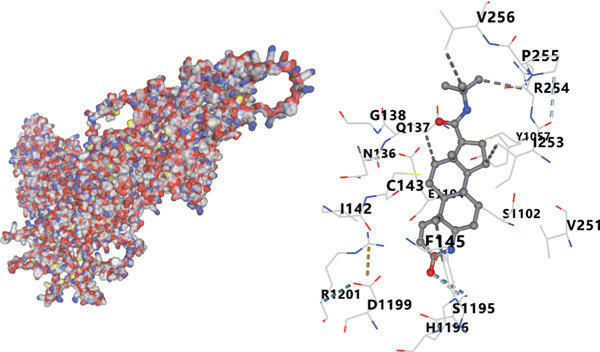
(d)

**Figure:**
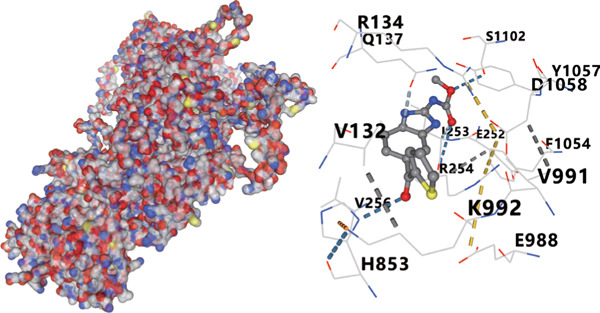
(e)

**Figure:**
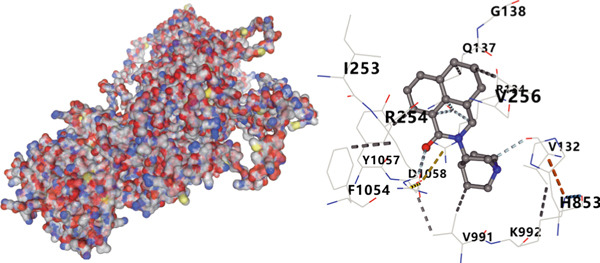
(f)

**Figure:**
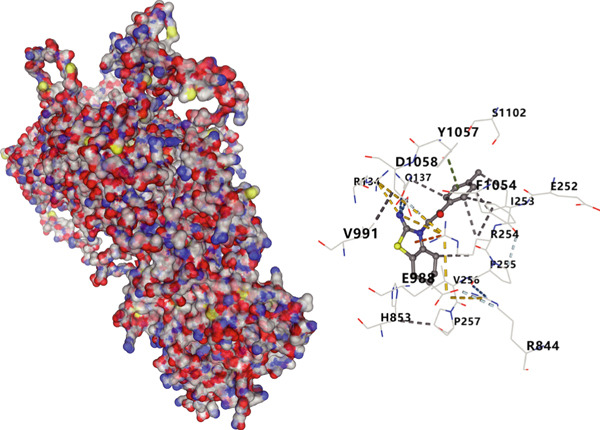
(g)

**Figure:**
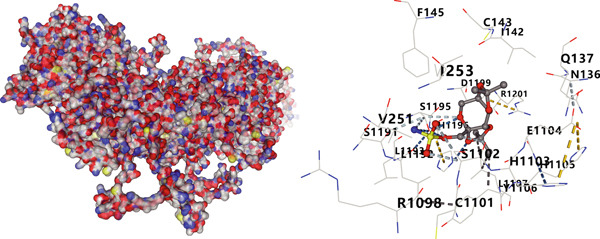
(h)

## 4. Discussion

KIRC, the most common histological RCC subtype, is characterized by poor prognosis and a tendency to metastasize. Despite recent advances in improving KIRC prognosis [30], further decreasing the mortality rate of KIRC remains a major challenge. Understanding related cellular and molecular mechanisms is essential to establish new clinical prognostic models and identify new biomarkers. This study systematically investigated the heterogeneity and key genes of immune cell subsets in KIRC by integrating single‐cell, GWAS, TCGA‐KIRC queue, GEO, and ArrayExpress data. Moreover, we built a clinical prognostic model based on T cell associated genes. Our results enhance understanding of the KIRC immune microenvironment and may guide new research directions in personalized therapy.

First, we identified seven major immune cell subpopulations in KIRC through single‐cell data analysis. The TRSs of T cell subsets in scPagwas analysis were substantially greater than those of other cell subsets, thus indicating T cells′ important roles in KIRC occurrence and related immune regulation. Immune cells play crucial roles in the context of the TME [31]. T cells are an immune system cornerstone with crucial functions in identifying and eradicating pathogens, as well as regulating abnormal cell growth [32]. The immune microenvironment coordinates T cell activity through multiple mechanisms. Tregs act as inhibitory immune cells within the TME, thereby impeding other immune cellular activity, such as by inhibiting T cell responses [33]. Moreover, immunosuppressive entities within the TME may hinder the normal function of T cells. T cell infiltration has been associated with negative outcomes in KIRC prognosis [34]. Therefore, further exploration of how T cells function in KIRC′s immunological microenvironment and in‐depth study of T cell‐associated immune regulatory mechanisms are expected to explain tumor progression approaches and lay a foundation for innovative therapeutic strategies for KIRC.

We conducted intersection analysis on key genes of T cell subsets, DEGs, WGCNA module core genes, and genes with PCC values > 0.05 in single‐cell analysis and obtained 86 significantly correlated intersecting genes with T cells. Functional enrichment analysis of these genes indicated their involvement primarily in signaling pathways associated with the immune response, leukocyte activation, cell adhesion, and immune escape, such as the NF‐*κ*B signaling pathway, TNF signaling pathway, and NOD‐like receptor signaling pathway. These pathways play crucial roles in the formation of the tumor immune microenvironment. The NF‐*κ*B signaling pathway is a key regulatory factor in immune response and inflammation [35–38]. NF‐*κ*B is an important signaling transcription factor that regulates various cellular processes, thus promoting proliferation, regulating cell death, stimulating migration, and mediating inflammatory processes, according to cell type, developmental stage, and pathological status [39, 40]. In KIRC, abnormal activation of the NF‐*κ*B signaling pathway drives tumor occurrence, progression, and treatment resistance through multiple molecular mechanisms. Peng et al. have reported that CARD10 knockdown inhibits activation of the NF‐*κ*B signaling pathway in RCC cells [41]. Vascular endothelial growth factor (VEGF) is a downstream growth factor in the NF‐*κ*B signaling pathway. Phospholipase C*ε* promotes the growth of RCC cells through NF‐*κ*B‐mediated upregulation of VEGF [42]. Regarding treatment resistance, curcumin may endow RCC with radiosensitivity by inhibiting NF‐*κ*B activation and its downstream rules [43]. Triptolidenol inhibits the growth of RCC by targeting the ATP binding site of IKK*β* and disrupting the NF‐*κ*B/COX‐2 pathway [44]. These studies have collectively revealed the pleiotropy of NF‐*κ*B as a core regulatory node in KIRC.

We constructed a clinical prognostic model containing seven risk genes (CASP4, CCM2, CCNL2, DOCK8, LENG8, PABPN1, and TCIRG1) with 101 combinations of 10 machine learning algorithms. The model demonstrated high predictive ability in the TCGA‐KIRC, GSE29609, and E‐MTAB‐1980 queues, and the AUC values of the time‐dependent ROC curves indicated its favorable accuracy and robustness. Survival analysis indicated that patients in the high‐risk group had significantly poorer prognosis, whereas patients in the low‐risk group had clear survival advantages. This model not only provides a new tool for evaluating the prognosis of patients with KIRC but also further verifies the important role of T cell related genes in the immune microenvironment and clinical outcomes of KIRC. Furthermore, by using machine learning algorithms such as XGBoost and LightGBM, we identified that DOCK8 made the greatest contribution to the model. DOCK8 is expressed and has been studied primarily in immune cells, and it plays key roles in cell migration, immune response activation, and T cell survival [45, 46]. DOCK8 deficiency can lead to combined immunodeficiency disease, which is clinically associated with chronic infections of multiple microbial pathogens; affects the number and migration of immune cells; and increases cancer risk [47–49]. The DOCK8 gene and epigenetic inactivation participate in cancer development by interfering with cell migration, morphology, adhesion, and growth [50]. Zhang et al. have found that high expression of DOCK8 indicates a favorable prognosis for HPV positive patients with HNSCC, as well as elevated immune infiltration in the microenvironment [51]. In addition, Gutierrez‐Ruiz et al. have reported that DOCK8 depletion significantly decreases tissue protease‐dependent extracellular matrix degradation and impairs invasive ability in PDAC cells [52].

Furthermore, in the functional enrichment analysis of low‐ and high‐risk groups, we observed significant differences in metabolism and immune pathways. In low‐risk patients, metabolic pathways such as fatty acid metabolism, propionate metabolism, and retinol metabolism were significantly enriched and therefore might have protective roles in inhibiting tumor malignant progression. In contrast, pathways such as cell adhesion molecules, dilated cardiomyopathy, and ECM–receptor interactions were significantly enriched in high‐risk patients, and consequently might exacerbate disease progression by promoting tumor infiltration and metastasis. In addition, the immune microenvironment in high‐risk patients exhibited notable immunosuppressive features, such as significant upregulation of Tregs. In KIRC, Tregs mediate immune suppression through various mechanisms and promote tumor immune escape. KIRC cells secrete inhibitory cytokines such as IL‐10 and TGF‐*β*, which in turn inhibit immune cell activation and promote Treg differentiation. Moreover, Tregs inhibit the activity of effector T cells and promote tumor growth [53]. Activated Tregs also induce cell death of CD4+ and CD8+ effector T cells through a granzyme‐dependent pathway [54, 55]. These results suggest that Tregs might serve as an important target in KIRC immunotherapy. Our results further support the value of the risk score as an independent predictor of clinical outcomes in patients with KIRC.

Although this study revealed the key molecular mechanisms underlying the occurrence and progression of KIRC, and constructed a clinical prognostic model with high predictive value, it has several limitations. First, because this study was based on publicly available datasets with limited sample sizes, further validation is needed in larger independent cohorts. Second, the functions of key genes in clinical prognostic models must be further validated through in vivo and in vitro experiments. Then, the results of drug sensitivity and molecular docking analysis must be validated for clinical feasibility in subsequent experiments. Finally, future research should include in‐depth genetic and epigenetic analysis of the DOCK8 gene to clarify whether its genetic changes drive its expression changes and functional abnormalities, and explore its interaction with existing driver gene mutation pathways, in order to more fully evaluate its potential as an independent biomarker or therapeutic target.

## 5. Conclusion

In summary, this study systematically analyzed the immune microenvironment, identified key genes, and developed a prognostic model for KIRC. The findings revealed the core roles of T cell subsets in the immune microenvironment of KIRC and indicated the effectiveness of our clinical prognostic model. Our research might provide new ideas and potential targets for the precise diagnosis and personalized treatment of KIRC. In addition, the discovery of DOCK8 as a key gene might become an important direction for the development of targeted therapies. Future research should combine experimental validation and clinical trials to further promote the translation of these findings.

## Author Contributions

X.Y., Z.J., and J.W. made equal contributions to this study. Y.K., X.H., and Y.H. jointly designed and supervised the study. X.Y., Z.J., J.W., and Y.Y. conducted and validated the bioinformatics analyses. X.Y., Z.J., J.W., S.W., and Y.H. designed the experimental protocols and performed the experiments. X.Y., Z.J., J.W., Y.K., X.H., and Y.H. drafted and revised the manuscript. X.H. and Y.H. provided financial support for publication.

## Funding

This study was supported by Natural Science Foundation of Jiangsu Province (10.13039/501100004608, BK20241796).

## Disclosure

All authors contributed to this article and approved the submitted version.

## Ethics Statement

This study was approved by the Ethics Committee of The First Affiliated Hospital of Soochow University with the number of 2025‐732 and conducted in strict accordance with the Declaration of Helsinki. All patients or their families signed informed consent documentation before sample collection.

## Conflicts of Interest

The authors declare no conflicts of interest.

## Supporting information


**Supporting Information** Additional supporting information can be found online in the Supporting Information section. Table S1: The detailed clinical data for the six pairs of KIRC tissues.

## Data Availability

The article includes the original contributions made in the study; any further questions may be addressed to the corresponding authors.
